# Pre-evacuation Time Estimation Based Emergency Evacuation Simulation in Urban Residential Communities

**DOI:** 10.3390/ijerph16234599

**Published:** 2019-11-20

**Authors:** Jiayan Chen, Jia Yu, Jiahong Wen, Chuanrong Zhang, Zhan’e Yin, Jianping Wu, Shenjun Yao

**Affiliations:** 1 School of Environmental and Geographical Sciences, Shanghai Normal University, Shanghai 200234, China; 1000441674@smail.shnu.edu.cn (J.C.); jhwen@shnu.edu.cn (J.W.); zhaneyin@shnu.edu.cn (Z.Y.); 2 Department of Geography, University of Connecticut, Storrs, CT 06269, USA; chuanrong.zhang@uconn.edu; 3 Center for Environmental Science and Engineering, University of Connecticut, Storrs, CT 06269, USA; 4 Key Laboratory of Geographic Information Science (Ministry of Education), East China Normal University, Shanghai 200241, China; jpwu@geo.ecnu.edu.cn (J.W.); sjyao@geo.ecnu.edu.cn (S.Y.)

**Keywords:** pre-evacuation time, predictive model, evacuation simulation, residential community, Random Forest algorithm

## Abstract

The timely and secure evacuation of an urban residential community is crucial to residents’ safety when emergency events happen. This is different to evacuation of office spaces or schools, emergency evacuation in residential communities must consider the pre-evacuation time. The importance of estimating evacuation time components has been recognized for approximately 40 years. However, pre-evacuation time is rarely discussed in previous community-scale emergency evacuation studies. This paper proposes a new method that estimates the pre-evacuation time, which makes the evacuation simulation in urban residential communities more realistic. This method integrates the residents’ pre-evacuation behavior data obtained by surveys to explore the influencing factors of pre-evacuation time and builds a predictive model to forecast pre-evacuation times based on the Random Forest algorithm. A sensitivity analysis is also conducted to find the critical parameters in evacuation simulations. The results of evacuation simulations in different scenarios can be compared to identify potential evacuation problems. A case study in Luoshanqicun Community, Pudong New District, Shanghai, China, was conducted to demonstrate the feasibility of the proposed method. The simulation results showed that the pre-evacuation times have significant impacts on the simulation procedure, including the total evacuation time, the congestion time and the congestion degree. This study can help to gain a deeper understanding of residents’ behaviors under emergencies and improve emergency managements of urban communities.

## 1. Introduction

Emergency management is crucial to preventing potential risks in urban communities [1]. In high-density urban communities, efficient, safe and timely evacuation is important for guaranteeing the safety of residents in emergencies [2,3].

Various emergency evacuation studies have been done all over the world. Many researchers conducted small-scale evacuations inside single buildings [4,5,6], while many other scholars developed evacuation optimization models on large-scale evacuations as well [7,8,9]. However, only a few studies have attempted to analyze emergency evacuations in urban residential communities with a consideration for residents’ behaviors from a microscopic perspective. Most of the previous studies have concentrated on risk assessments of evacuations [10,11] or personnel evacuation simulations within buildings [12,13]. Due to the complexity of resident structure in urban communities, residents may have various behavioral responses to emergencies. Moreover, members in a family focus on each other to ensure the safety of family members [14,15,16]. Therefore, the evacuation processes in communities is quite different from the evacuations in office buildings, subway stations and other public places.

Previous studies in emergency evacuations have provided evidence that some occupants may not evacuate immediately and attempt to confirm and evaluate the risk of emergency events [17]. Moreover, people may fight a fire or help others [18,19] when encountering an emergency, such as a fire. The importance of estimating the components of evacuation time, like authorities’ decision time, warning dissemination time, household preparation time, and evacuation travel time, has been recognized for approximately 40 years. Urbanik et al. [20] proposed a basic formulation and Urbanik [21,22] updated it. Lindell and Perry [23] and Lindell and Prater [24] critiqued transportation engineers assumptions about evacuation time components. Lindell et al. [25] summarized the state-of-the-art in large scale evacuations and Lindell et al. [26] reported empirical departure times for a flash flood evacuation. There is also empirical data from tsunami evacuation studies by Blake et al. [27], Goto et al. [28], Lindell et al. [29], and Sun et al. [30,31,32]. Although little attention has been paid to evaluating the pre-evacuation time, the issue of community evacuations was studied by Lindell et al. [25]. Lindell et al. [26] addressed the predictors of evacuation delay from a flash flood. This study indicated that pre-evacuation delays often occurred in evacuations due to the differences in behavioral responses to emergencies, which may be influenced by the factors relating to characteristics of occupants, buildings and emergencies [33,34,35].

Furthermore, due to the urgency and complexity of emergencies, the evacuation processes have a great deal of uncertainty. However, some possible situations of an evacuation have been neglected in traditional evacuations, particularly in the estimation of evacuation times. The neglect may cause inaccuracy in estimating personal evacuation times and the total evacuation time. To reduce the uncertainty, parameter sensitivity analysis is a critical step in determining values of important parameters, which have a significant impact on the simulation results. Among existing methods of sensitivity analysis, the one-at-a-time (OAT) method has been commonly used [36,37]. This method has a simple calculation procedure and is easy to be understood. It is suitable for conducting a parameter sensitivity analysis for evacuation simulations.

People’s responses to emergencies are generally based on the effect of psychology, evacuation environment, etc. However, people’s behavior intentions largely determine actual behaviors according to the theory of reasoned action (TRA) [38]. Meanwhile, general research findings might not permit researchers to conduct specific studies about emergency management for specific behaviors of residents in communities. A survey demonstrated a significant degree of correspondence between respondents’ evacuation expectations and actual behaviors [39]. Behavioral expectations have a limitation in that it cannot assess people’s moment-to-moment responses to actual evacuation situations or warning messages, behavioral tendencies and perceptional responses on evacuation decisions, which they have not previously encountered. However, actual evacuation studies and hypothetical evacuation studies identify the same predictors of evacuation decisions. A lot of similarity between responses to hypothetical scenarios and those of actual evacuations were indicated by Huang et al. [40]. Based on the behavioral expectations and actual evacuation behavior studies, the questionnaire in this study was conducted on the basis of behavioral expectations.

Considering the limitations of existing studies, a new evacuation simulation method, which considers the pre-evacuation times of residents in urban residential communities, is proposed in this study. This paper tries to address an evacuation problem in a vertical building evacuation followed by a small-scale horizontal pedestrian area evacuation. In this method, a predictive model based on the Random Forest (RF) algorithm was built to forecast pre-evacuation times. It was able to conduct the training and verification of pre-evacuation behavioral information obtained by questionnaires and estimate the delay times for different residents. The method also integrated indoor and outdoor evacuation processes. A case study in Pudong New District, Shanghai, China was conducted to demonstrate the feasibility of the method. With the evacuation simulation result (including the total evacuation time, the congestion time and the congestion degree), analysis in the study area and a comparison with the scenario, which did not consider pre-evacuation time, the advantage of this method was demonstrated.

## 2. Methodology 

**Figure 1:**
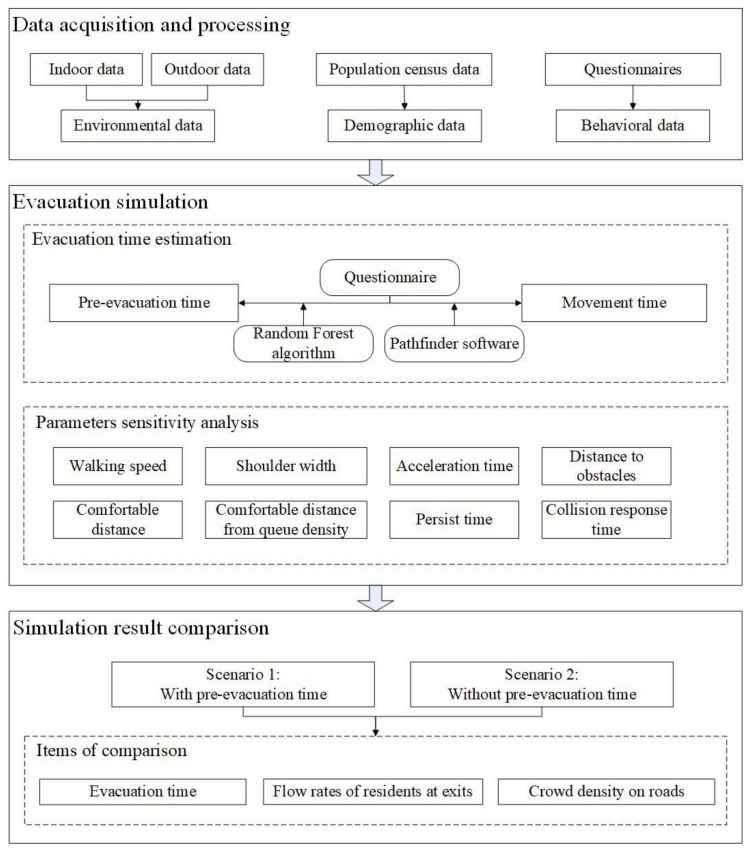
Framework of the proposed method for evacuation simulations based on pre-evacuation time estimation.

Figure 1 illustrates the framework of the proposed method, which describes the evacuation simulation procedure. There are three parts of the framework as follows:

### 2.1. Data Acquisition and Processing

#### 2.1.1. Environmental Data

Environmental data were mainly obtained to construct the evacuation space during emergency evacuation simulations, which included two parts: indoor data and outdoor data.

##### (1) Indoor Data

Indoor data comprised of house types, corridors and stairways. These data was obtained from real estate companies or construction planning bureaus. Due to the characteristics of buildings such as floors, materials and electrical equipment, evacuations within buildings are more complex than the outside, particularly in high-rise buildings [41]. The human behaviors in buildings is one of the key factors affecting the evacuation time [42].

##### (2) Outdoor Data

Outdoor data included community roads, building layouts and exits that was acquired from community layout plans and aerial images. The layout of buildings in a community can directly affect the spatial distribution of residents. Moreover, the layout of the road network also has significant impacts on the efficiency of evacuation [43].

#### 2.1.2. Demographic Data

Demographic data, including gender ratio and the age structure of residents, had a significant impact on the results of evacuation simulations. The residential population distribution data was generated from the population census data or the actual population data acquired by field surveys.

#### 2.1.3. Behavioral Data

The evacuation behavior is an important factor, which affects the evacuation time. For instance, Wang et al. [44] described the individual level data needed for a pedestrian evacuation model of an environmental hazard (tsunami). Evacuation behaviors are very complex and they are reflected mainly in the pre-evacuation time and route planning. Compared to emergency evacuations in schools, office buildings, supermarkets or other public spaces, emergency evacuations in residential communities involve different types of residents and complex social relationships. Pre-evacuation behavioral data was collected using questionnaires. Respondents had to answer questions focused on personal information and pre-evacuation behaviors. The questionnaire is presented in Appendix A for reference, including the following information:

(1) Basic personal information, including gender, age, education, physical condition, family members, evacuation training experience and so on.
(2) Personal behavior at the initial stage of an emergency, including the first reaction when an emergency occurs, and his/her estimation of the pre-evacuation time.
(3) Personal behavior during an evacuation, including the choice of stairways and the route selection when congested.

### 2.2. Evacuation Simulation

In general, there are two main types of emergency evacuation simulation methods: evacuation drills and computer-based evacuation simulations. Evacuation drills are reliable methods and the results are more realistic. However, it is costly to utilize such time-consuming and laborious methods to conduct evacuation simulations with tracking and recording the behaviors and trajectories of evacuees. Moreover, evacuation behaviors are quite complex and may be different even in the same scenario, which greatly affects route selection, evacuation time and efficiency [45]. Therefore, in this study the computer-based evacuation simulation method was chosen, which used a typical simulation software, Pathfinder, to conduct emergency evacuation simulations. 

Pathfinder is an agent-based egress and human movement simulator. The evacuation simulation of Pathfinder provides two primary modes: SFPE mode and Steering mode, and a number of virtual residents can be set up based on complex evacuation rules to walk. An evacuation simulation environment can be built in a graphical interface, including the layout of community, floors, stairways, entrances and exits of buildings. Once the environment is built, properties of residents can be set, such as walking speed, shoulder width, acceleration time, and some behavior characteristics such as pre-evacuation time and designation points. After these basic parameters have been set the evacuation simulation can be conducted.

Because various factors can affect evacuation times in realistic situations, it is necessary to make several assumptions to simplify the scenario of evacuation simulation. Firstly, the delays due to authorities’ decision time and warning dissemination time were not considered in this study. Secondly, to eliminate common causes of delays such as reuniting separated family members in departure time [26,46], the time devoted to evacuation was a weeknight, when all residents were at home before the evacuation. The third assumption was that we did not consider complex conditions such as incomplete compliance and evacuation shadow [26], incomplete warning reception, attention, and comprehension, as well as physical mobility limitations [47,48]. The last assumption was that no one was overtaken by the hazard, such as a wildfire and tsunami.

### 2.3. Evacuation Time Estimation

Evacuation time is one of the most important factors that measures the efficiency of evacuation. Relevant studies believe that saving evacuation time is the primary goal in evacuation optimization [49,50,51]. Due to the uncertainty of emergencies, it poses a great threat to personnel safety if residents take a long-term detention. In this study, the evacuation time was divided into two parts, these were pre-evacuation time and movement time. The pre-evacuation time was related to personal characteristics, psychology, disaster experience, social relations and so on. Simultaneously, the movement time depended on many factors, such as the crowd density, personal walking speed, and the widths of exits. In general, the total evacuation time can be calculated using the following equation:

(1) $$ET=T_{pre}+T_{mov}$$

where ET is the total evacuation time; $T_{pre}$ is the pre-evacuation time which represents the time interval between the time of receiving a warning and the beginning of movement; $T_{mov}$ represents the movement time during evacuation, that is, the time from leaving home to reaching the safe place.

To calculate $T_{pre}$ accurately, a predictive model based on the Random Forest (RF) algorithm was built to predict the pre-evacuation time based on behavioral characteristics of residents. The RF algorithm is a machine learning algorithm proposed by Leo Breiman [52], which can be regarded as an extension of Breiman’s bootstrap aggregation idea [53] and is inspired by random subspace [54] and earlier work about shape classification [55].

A RF is composed of multiple decision trees. A decision tree is a tree-like model that uses a branching method to illustrate every possible outcome of a decision. The tree consists of directed edges and nodes, including a root node, branch nodes, and leaf nodes. The root node is an aggregation of training data. The branch nodes represent the principles of classification. The data which arrive at the branch nodes will be classified by rules. Leaf nodes represent the final classification results. Each path from the root node to a leaf node represents a classification process.

This study assumes an unknown connection relation between the p-dimensional input vector $x={\left(x_{1},\ldots ,x_{p}\right)}^{T}$ and the output variable y. The set of possible values of y is denoted by Y. The goal is to predict y based on the classification models with the variable x.

**Figure 2:**
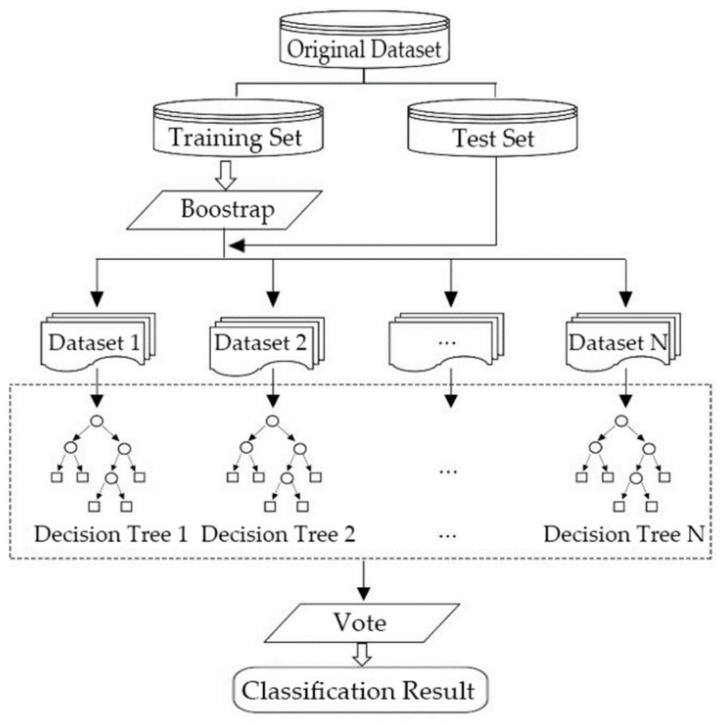
Architecture of the Random Forest algorithm.

The training and classification process of the RF algorithm includes three steps shown in Figure 2:

(1) Using the Bootstrap method to take samples for N times from the training set to generate N datasets after dividing the original dataset into the training set and test set. 

(2) Constructing N decision trees as classification models $\left\{h_{1}\left(x\right),h_{2}\left(x\right),\ldots ,h_{N}\left(x\right)\right\}$ to classify N datasets. For the nth tree, a random vector $\theta_{n}$ is generated. On the condition of sufficient trees, $h_{n}\left(x\right)=h\left(x,\theta_{n}\right)$. A set of models depend on the values of $\theta_{n}$ can be presented as follows:

(2) $$\left\{h\left(x,\theta_{n}\right),n=1,2,\ldots ,\;N\right\}$$

where n is the nth tree; N is the number of trees; $\theta_{n}$ is the random vector which is independently distributed with $\theta_{1},\ldots ,\theta_{n-1}$ but with the same distribution.

N classification results will be generated in this step, respectively.

(3) Voting the N classification results and using the most frequently predicted class as the final output result without pruning of the decision trees. The decision for voting is shown in Equation (3):

(3) $$H\left(x\right)=arg\underset{y\in Y}{\max}\overset{N}{\underset{n=1}{\sum }}I\left(h_{n}\left(x\right)=y\right)$$

where $H\left(x\right)$ is an ensemble classification model, which represents the most frequently predicted class; $h_{n}\left(x\right)=y$ means the prediction of the variable y using the nth tree with the variable x; $I\left(\cdot \right)$ is the indicator function.

With the aforementioned voting procedure, the final classification result can be generated.

In this study, the objective of using the RF algorithm was to predict the pre-evacuation time of residents based on the individual information of residents. Questionnaires were designed to collect residents’ information for surveying pre-evacuation behaviors. Some questions were set as input variables and the answer about pre-evacuation time was chosen to be the output variable in RF. The major questions involved in the prediction model are listed in Table 1.

After estimating the pre-evacuation time, the movement time in evacuation ($T_{mov}$ in Equation (1)) was also measured by evacuation simulations. The evacuation simulation software Pathfinder was utilized to simulate the emergency evacuation process of residents. 

### 2.4. Parametric Sensitivity Analysis

Due to the urgency of emergency events and the complexity of the evacuation procedure, the estimation of evacuation time involved some uncertain parameters in evacuation simulations, including the spatial distribution and attributes of residents, such as walking speed and shoulder width.

Under most circumstances, it is impossible to conduct an enormous amount of evacuation drills to analyze the uncertainty in evacuation. Meanwhile, value setting of critical parameters cannot be determined by subjective judgments or default values in evacuation software. In such a case, sensitivity analysis methods can deal with parameter uncertainty problems effectively. Typically, it is one of the key modules in most model development processes [56,57], which can reduce the complexity of a model by describing how important the input variables are to the output variables quantitatively.

In the research field of sensitivity analysis, various kinds of methods have been proposed, such as Monte Carlo simulation and one-factor-at-a-time (OAT). OAT is one of the most commonly used methods, which has a simple calculation procedure and is easy to be understood. OAT-based parameter sensitivity analysis examines the influence of the perturbation of a single parameter on the output, while others remain constant. It can determine which input parameters $x_{i}$ have greater impacts on the output result y by observing the influence of perturbation of parameter values on the result. The degree of influence can be expressed as the un-normalized sensitivity index $US\left(x,y\right)$ as follows [58]:

(4) $$US\left(x,y\right)=\frac{\partial y}{\partial x_{i}}$$

where $x_{i}$ is the ith input parameter and y is the output result.

Based on Equation (4), the normalized sensitivity index$\;NS\left(x,y\right)$ [59] can be utilized to eliminate the difference in input parameters $x_{i}$ with different units and magnitudes. The equation of the normalized sensitivity index can be expressed as follows:

(5) $$NS\left(x,y\right)=\frac{\partial y/y}{\partial x_{i}/x_{i}}=\frac{x_{i}\partial y}{y\partial x_{i}}\approx \frac{x_{i}\Delta y}{y\Delta x_{i}}$$

According to the definition of the partial derivative, the normalized sensitivity index was only applicable to the small variation ($\Delta x_{i}$) in the input parameter. Therefore, the method of local sensitivity analysis was used in this study, that is, adding a stepwise percentage change on the value of input parameters, and then the effect on the output was observed [60].

The sensitivity index $S\left(x_{i,n},y_{i,n}\right)$ can be calculated by the following equations:

(6) $$S\left(x_{i,n},y_{i,n}\right)=\frac{x_{i,n}}{\Delta x_{i}}∕\frac{y_{i,n}}{\Delta y_{i,n}}$$

(7) $$\Delta x_{i}=x_{i,n}-x_{i,0}$$

(8) $$x_{i,n}=x_{i,0}\times \left(1+\mathrm{IPC}\times {\mathrm{step}}_{n}\right),\;{\mathrm{range}}_{\min}\;\leq \;{\mathrm{step}}_{n}\;\leq \;{\mathrm{range}}_{\max}$$

where $x_{i,n}$ is the nth percentage changed value of the ith input parameter; $\Delta x_{i}$ is the increment/decrement of value change of the ith input parameter compared with the original value of this parameter; $y_{i,n}$ is the output result (the total evacuation time in this study) with the nth percentage value change of the ith input parameter; $\Delta y_{i,n}$ is the value change of the output result when compared with the original result when making the nth percentage value change of the ith input parameter; $x_{i,0}$ is the original value of the ith input parameter; IPC is the increment of percentage change (10% in this study); ${\mathrm{step}}_{n}$ is the nth integer value ranging from range_min_ to range_max_; range_min_ and range_max_ were −5 and 5 in this study, respectively. With different ${\mathrm{step}}_{n}$, stepwise percentage change on the value of input parameters could be conducted and the OAT sensitivity analysis could be carried out.

### 2.5. Simulation Result Comparison

In this study, two different scenarios were preset for evacuation simulations. Scenario one considered the pre-evacuation behaviors of residents, while Scenario two neglected the pre-evacuation times and all the residents started to be evacuated at the same time. With the evacuation simulations of these two scenarios, their simulation results were compared to quantitatively analyze the impact of pre-evacuation time on the evacuation procedure. The comparison had different items, including “evacuation time”, “flow rates of residents at exits” and “crowd density on roads”. With such a detailed comparison, the significance of pre-evacuation estimation for the evacuation simulation in a community can be demonstrated. 

## 3. Case Study

### 3.1. Study Area

**Figure 3:**
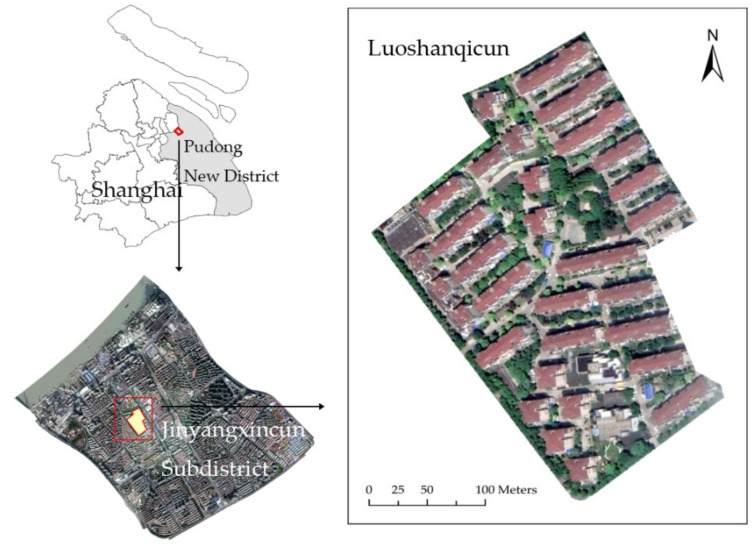
Location of Luoshanqicun Community, Jinyangxincun Subdistrict in Pudong New District, Shanghai, China.

A case study in Luoshanqicun, which is a residential community located in Pudong New District, Shanghai, China, was made to verify the feasibility of the method (Figure 3). The Luoshanqicun Community was built in 1996 with an area of 49,000 m^2^ and is affiliated to Jinyangxincun Subdistrict and located not far from the Huangpu River. Because this community was built over 20 years ago, it is quite different to the new communities in Shanghai. The stairways of the buildings have relative narrower widths. Moreover, there are only two exits located in the Southwest and Southeast of the community. The current situation of the study area was suitable for our case study.

### 3.2. Data Processing and Parameter Setting

#### 3.2.1. The Layout of the Community

The study area had 51 six-story residential buildings, including 1,284 apartments. We divided the spatial data of the study area into two parts: indoor data and outdoor data. The main indoor structure included apartments, corridors and stairways in the buildings. Outdoor data included the community roads, building layouts and the locations of exits. All the building data were acquired through the field measurements and drawn by AutoCAD and Pathfinder software.

#### 3.2.2. Resident Data

##### (1) Demographic Data

According to the data of the Sixth Population Census of China, the demographic data at the scale of the community were obtained. The data included gender, age, educational level and other personal information of the residents. 

There were 4668 residents, including 3964 permanent residents and 704 external residents, 2254 males and 2414 females living in the study area. The ages of residents were divided into four groups. There were 456 elderly people over the age of 65 years and 390 children under 13 years. According to the statistics, more than 45% of residents were adults between 36 and 64 years.

##### (2) Residents’ Pre-evacuation Behavioral Data

After residents receive the alarm signal, they will take some actions before evacuation. Pre-evacuation behaviors have a great impact on the total evacuation time. Awareness and behaviors in the emergency of residents with different genders, age, education and living conditions may have remarkable differences. Therefore, this study conducted a survey of pre-evacuation behaviors by means of questionnaires in the community. The hazard scenario for the questionnaires respondents was an earthquake. Compared to a typhoon or a hazardous materials release, an earthquake may have obvious environmental cues which can provide sufficient warning of the threat of building collapse. Therefore, any delay due to authorities’ decision time and warning dissemination time was not considered in this case study. The proportions of respondents of different age and gender groups in the questionnaires were set according to the age and gender structure in the community, which was obtained from the census data (Table 2). The questionnaire (Appendix A: Table A1) was distributed to the residents in the community with the help of the community managers and the respondents were interviewed face-to-face. Meanwhile, to get more samples of residents’ behaviors in the pre-evacuation stage, the questionnaire was also posted online. A total of 450 interviews were performed. After rejecting the unqualified questionnaires which lacked logicality and content integrity, 379 valid questionnaires were obtained, including 192 interviewed questionnaires and 187 electronic questionnaires. 

The data acquired from these questionnaires were set as the original dataset of RF (Figure 2) to predict the pre-evacuation times. The pre-evacuation behaviors were categorized into six classes in this study (Table 3). A resident may have had one or more behaviors listed in this table.

**Figure 4:**
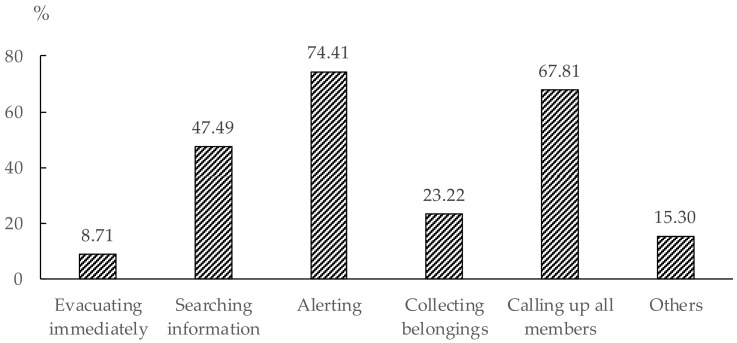
Statistical result of the selection of different pre-evacuation behaviors.

Based on the data collected from the questionnaires, we counted the selection of "Evacuating immediately" and other classes, respectively. Figure 4 shows that about half of the residents selected ‘Searching information’ to reassess the authenticity and criticality of emergencies by themselves, while only 8.71% of respondents expressed the willingness to evacuate immediately. 74.41% of residents selected ‘Alerting’ indicating that they had alerted others directly when they noticed warnings. These residents were concerned for the safety of others, such as the members of their family, roommates or neighbors. 67.81% of respondents selected the ‘Calling up all members’ option. Most of them lived with members of their family. They chose to evacuate together with others. This behavior was called ‘social attachment’ [61]. 15.3% of respondents stated that they would perform other activities except for the pre-evacuation behaviors mentioned above before an evacuation. 61.74% of respondents selected two or three behaviors simultaneously. Among them, 29.55% had two pre-evacuation behaviors, and 32.19% had three. 12.93% of the respondents selected four behaviors. 3.69% of these respondents selected five behaviors. These respondents thought that they needed to set aside more time than others to complete pre-evacuation behaviors.

**Figure 5:**
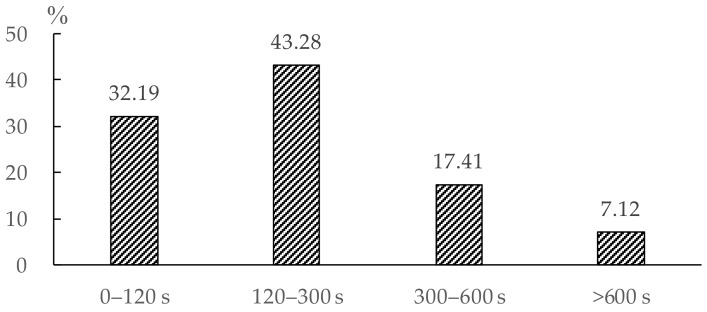
Estimated pre-evacuation time.

In this case study, the pre-evacuation time of a resident was defined as the delay time starting from the alarm being raised and ending with the evacuation of the resident. However, it was difficult to estimate the pre-evacuation time accurately just by subjective judgment. Therefore, the pre-evacuation times of residents were intended to be estimated by the predictive model introduced in Section 2.3 based on the questionnaires. The pre-evacuation time of a respondent was the delay time for completing all of his/her pre-evacuation behaviors (refer to Table 3). The respondents had to estimate the time and fill in the questionnaire. We divided the pre-evacuation times, which were collected from all the respondents into four time intervals: 0−120 s, 120−300 s, 300−600 s and >600 s and conducted a statistical analysis of the questionnaires (Figure 5). Because the time intervals gave the ranges of times rather than the exact time, the exact pre-evacuation time was randomly generated for each resident in several sub-intervals in accordance with the frequency statistics of the questionnaire data. The detailed method is introduced in Section 3.2.3. According to the statistical result, over 75% of respondents spent less than five minutes in the pre-evacuation period. Among them, 32.19% of residents chose to evacuate immediately within two minutes, while 43.27% chose to evacuate between 120 to 300 seconds after receiving the alarm signal. This result shows that most people tend to evacuate in a short period of time. However, 7.12% of respondents chose to evacuate after more than ten minutes, which might highly increase the evacuation risk in an emergency, such as an earthquake disaster.

#### 3.2.3. Prediction of Pre-evacuation Time

As mentioned in an earlier section, this study analyzed pre-evacuation behaviors and collected the pre-evacuation times of residents by conducting questionnaires and field surveys. We tried to estimate the pre-evacuation time of a resident by predicting the rules between personal information and pre-evacuation time based on the questionnaires and RF algorithm.

To build the predicting model, the data collected from questionnaires was first processed. The answers of questions (input parameters) in the questionnaire had variable forms, which could be binary variables or category variables. In the study, the ‘sequential method’ was used to standardize the value of the input variables and assign a unique code according to the sequence number for each option of a question involved in the questionnaire [62]. Some cases of the standardized input and output variables are listed in Table 4. The parameters of the prediction model are listed in Table 5.

By importing these input variables, the RF algorithm was utilized to estimate the relationship between multiple input variables and the output variable. Using a random function, such that each of the cases could be chosen with equal probability, we divided the original dataset into two sets. 70% of the cases were selected to generate the training set and 30% of the cases were chosen to produce the test set. There were 500 decision trees pre-set in the RF model to vote for a case simultaneously and judge the time interval to which it belonged. The time interval with the largest number of votes was the final result. For example, the first case had 465 decision trees, when judged it belonged to the second time interval (120−300 s). Therefore, the final output was 2, which was consistent with the case data in the test set. The overall classification accuracy of the RF algorithm in this study was 88.14%. It was much bigger than the base rate of 32.6%, which was computed by $\sum p{\left(Y_{i}\right)}^{2}$. A comparison between the classification accuracy of the RF algorithm and the base rate can demonstrate the advantage of the RF algorithm in classification accuracy.

Based on the model described above, the classification result, which represented the rules between personal information and pre-evacuation time was generated. Given the utilization of these classification rules, the personal data of all the residents in the study area were imported into the model to predict the pre-evacuation times. The predicted results are shown in Table 6. The column of “Predicted result (time interval)” was regarded as the predicted result of the pre-evacuation time for each resident in the community. The column showing “Actual response (time interval)” were the respondents’ actual responses, which were compared with the predicted results.

To demonstrate the advantages of the RF-based prediction model, we made a comparison between the predicted results of the RF algorithm and Back-Propagation (BP) neural network. The BP neural network model, which is one of the artificial neural network models has self-learning, self-adapting and generalization abilities [63]. It is widely used in classification, regression, fitting and other fields, which can deal with problems of linear and nonlinear input-output relationships. 

A comparison of the prediction performance between the RF algorithm and BP neural network is presented in Table 7. This table shows that the RF algorithm had a better predictive performance than the BP neural network.

Because the predicted pre-evacuation time of a resident only gave a relatively wide range of time when the resident would start to evacuate, we further divided the time interval into several sub-intervals. The statistical probability that a resident would be evacuated in a sub-interval time was calculated based on the frequency statistics of the questionnaire data. A random time was generated for each resident in accordance with this statistical probability and it was the final pre-evacuation time of this resident.

#### 3.2.4. Parameters of the Simulation Model

**Figure 6:**
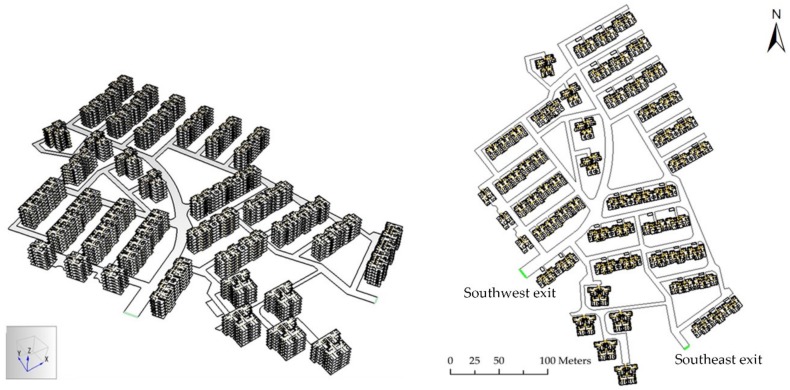
The oblique and plan view of the Luoshanqicun Community, Pudong New District, Shanghai, China.

The layout of the study area (oblique and plan view) is shown in Figure 6. In evacuation simulations, each resident needs to be pre-set a series of attributes. According to the experiences reported in previous research results, the values of these attributes can significantly affect the evacuation process [64,65,66,67]. This study set the average walking speeds and shoulder widths of residents with different sexes and age groups as fixed values. Table 8 shows the average walking speeds of different sexes and age groups [64,65]. The shoulder widths of different age-groups are also shown in Table 8 as well [66,67]. Furthermore, we set other parameters to default values on Pathfinder software.

### 3.3. Simulation Result

Based on the three-dimensional environmental data, demographic data and residents’ personal behavior data of the study area, an evacuation simulation process was conducted and the simulation result (Scenario 1) was generated. To quantitatively analyze the impact of pre-evacuation time on the evacuation process, a scenario which did not consider pre-evacuation time (Scenario 2) was also set to be compared with the simulation results. The simulation results and comparisons are presented in Table 9.

#### 3.3.1. Evacuation Time

Table 9 lists the evacuation times in two different scenarios for different locations, including the southwest exit, the southeast exit, the whole road network, and the doors of buildings. For example, there were 1,065 residents who were evacuated through the southwest exit with an average flow rate of 2.44 person/s in Scenario 1. Table 9 also shows that the last person took 483.6 s to be evacuated when the first took 47.3 s, and the total evacuation time of the Southwest exit was 436.3 s.

Compared with Scenario 2, the start time of the evacuation at the Southwest exit increased by 16.79% (6.8 s) when the end time increased by 0.29% (1.4 s) in Scenario 1. The total evacuation time within the building was shorter in Scenario 1 than in Scenario 2, which was reduced by 11.29% (44.7 s). Meanwhile, the evacuation time on the whole road network within the community increased by 18.99% (90.5 s) in Scenario 1.

#### 3.3.2. Sensitivity Analysis

**Figure 7:**
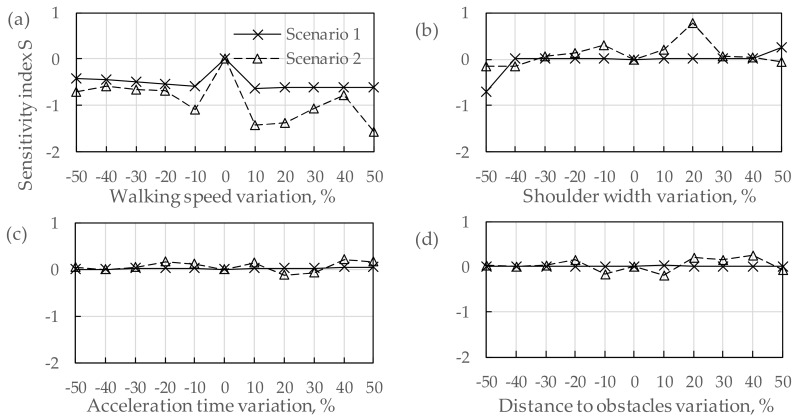

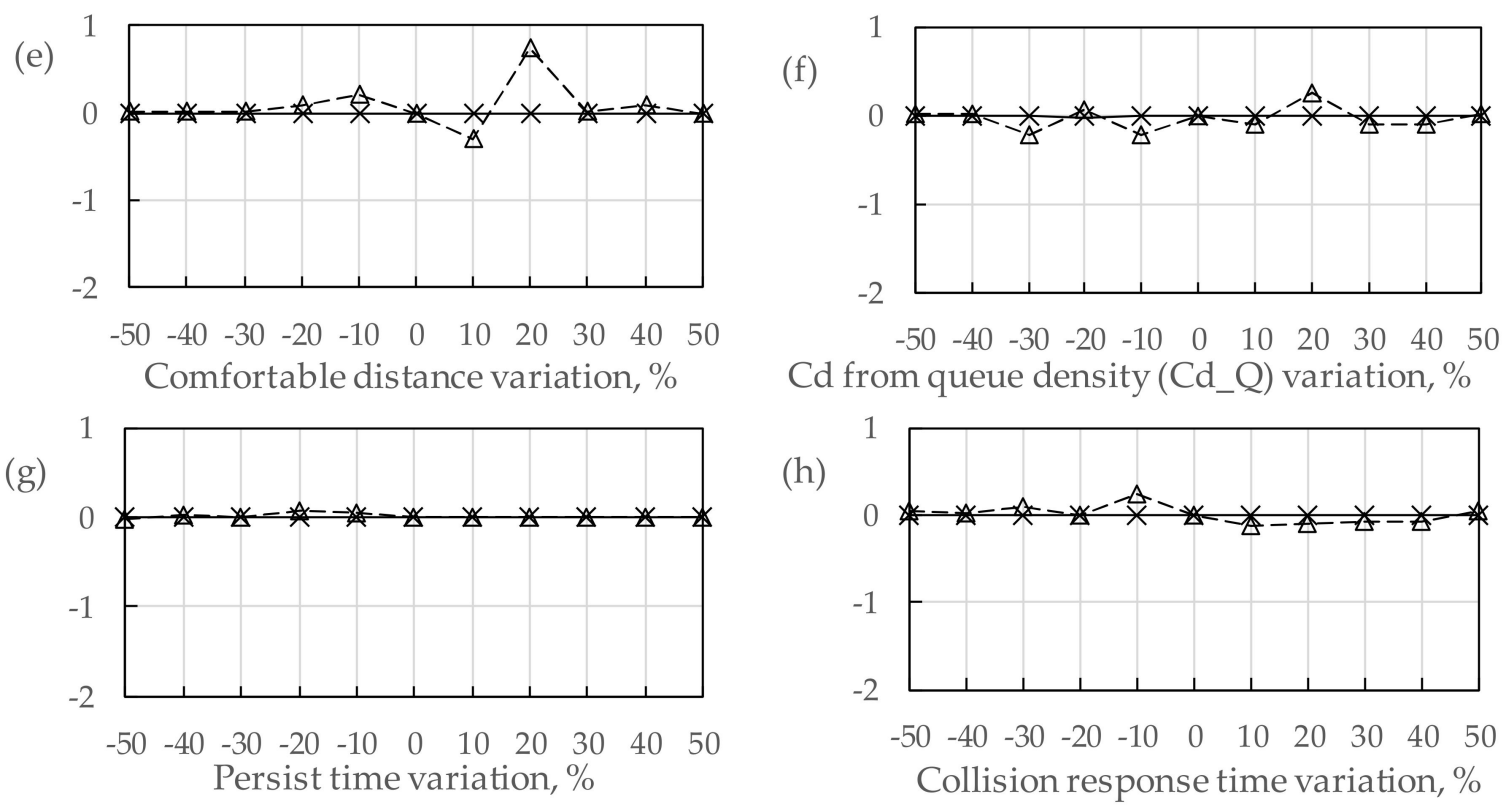
Sensitivity analysis of parameters assessed under two scenarios. (**a**)–(**h**): The sensitivity analysis results of different parameters, including walking speed, shoulder width, acceleration time, distance to obstacles, comfortable distance, comfortable distance from queue density, persist time and collision response time.

In this study, a sensitivity analysis was conducted to analyze the extent to which variation in each key parameter produced corresponding variation in the estimation of the total evacuation time. According to Equations (6)–(8), the study set a stepwise percentage change on the value of an input parameter, while all other parameters remained constant. Figure 7 gives the sensitivity analysis results, which shows the value changing of the sensitivity index according to the stepwise percentage change of different parameters. In this figure, it can be seen that different parameters had different sensitivity degrees as follows:

##### (1) Walking Speed (Ws)

Figure 7a shows that the value of the curve on the left side of the X-axis was smaller than zero. This illustrates that the evacuation time would be longer when the speed gets slower. Meanwhile, the value of the curve, which was on the right side of X-axis was also smaller than zero. This illustrates that the faster the speed is, the shorter the evacuation time. Judging from this sensitivity analysis result, minor changes in walking speed had a significant impact on evacuation time.

##### (2) Shoulder Width (Sw)

Figure 7b shows that the perturbation of the sensitivity index in Scenario 2 was obvious. The sensitivity index changed significantly if the changing ratio exceeded 20%. In Scenario 1, the simulation result was insensitive to the shoulder width and remained mostly stable. However, when the value of the shoulder width increased or decreased by more than 40%, the sensitivity was significant. This indicates that the sensitivity of the shoulder width cannot be neglected if the shoulder width has a larger or smaller value when considering pre-evacuation time.

##### (3) Other Parameters

Other parameters involved in the evacuation simulation included “acceleration time (At)”, “distance to obstacles (Do)”, “comfortable distance (Cd)”, “comfortable distance from queue density (Cd-Q)”, “persist time (Pt)” and “collision response time (Crt)”. In Scenario 2, Figure 7c–h shows that the influences of these parameters were relatively remarkable to the sensitivity index, especially “comfortable distance”. Minor value changes had a significant impact on the evacuation time for this parameter. However, in Scenario 1, the variations of these parameters had a small influence on the evacuation time. This means that, when simulating pre-evacuation behaviors and considering pre-evacuation time, it is expected that for “walking speed” and “shoulder width”, the sensitivities of most parameter values will not be significant to the evacuation time.

#### 3.3.3. Flow Rates of Residents at Exits

**Figure 8:**
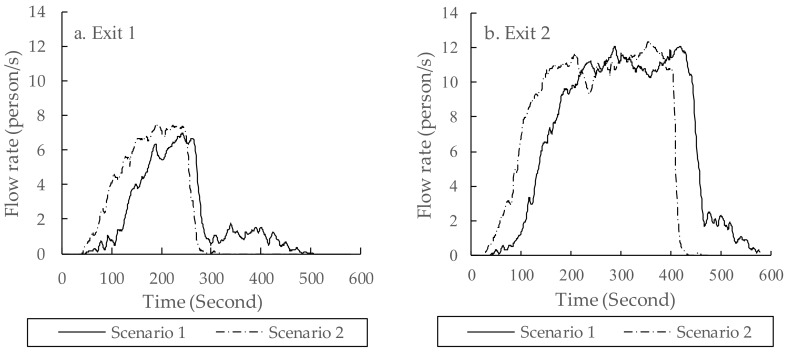
Flow rates of two exits under two scenarios. (**a**) The evacuation flow rate of the Southwest exit (exit 1); (**b**) the evacuation flow rate of the Southeast exit (exit 2).

Figure 8 shows the evacuation flow rates of the two exits in the community. The flow rate peaks and the times at which they occurred were specific to this study and had no general significance for evacuation modeling or evacuation planning. It can be seen from this figure that:

(1) The peaks of the flow rate appeared later under Scenario 1 in both panels of Figure 8, i.e., the flow rate distributions for Scenario 1 shifted to the right of the flow rate distributions for Scenario 2.

(2) At exit 1, the total evacuation time was greatly extended in Scenario 1 when compared with that of Scenario 2. It maintained a low flow rate for about 200 s after 300 s. Similarly, the time was extended at exit 2. Both panels of Figure 8 show that the flow rate distributions for Scenario 1 had longer tails than the flow rate distributions for Scenario 2. This was a result of pre-evacuation time being a distribution (as shown in Figure 5) rather than a constant (which would be the case if everyone were delayed for exactly the same amount of time).

#### 3.3.4. Crowd Density on Roads

**Figure 9:**
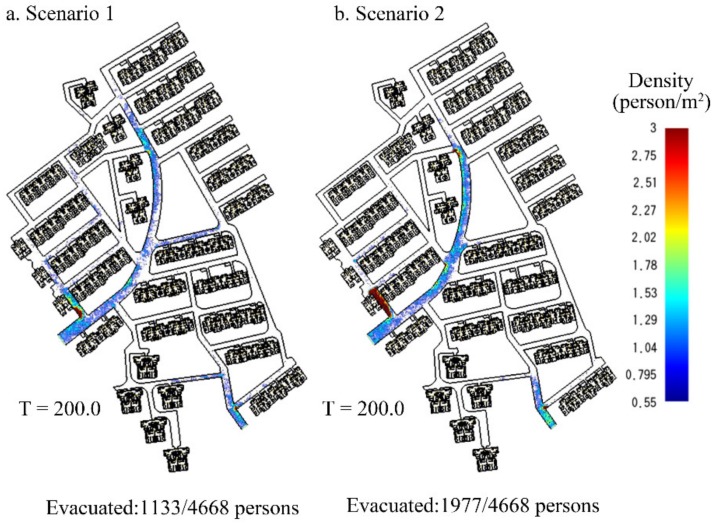
Crowd density of residents on roads in two scenarios. (**a**) Scenario 1: With pre-evacuation time. (**b**) Scenario 2: Without pre-evacuation time.

The density mapping of residents on the whole road network was a spatial measurement to evaluate the evacuation process. The mapping results were a series of crowd density maps of residents, which were relative to different moments in evacuation. Figure 9 shows an example of the crowd density maps at 200 s into an evacuation under the two scenarios. High densities appeared in certain areas near the two exits and major intersections. The accumulation of residents on the roads can easily produce congestion. This illustrates the point that congestion is likely to form where evacuation routes converge because the capacity of the downstream link is usually smaller than the sum of the capacities of the upstream links [68]. The region that had the highest crowd density was sideways near the Southwest exit. The highest density of this region was 2.389 person/m^2^ in Scenario 1, which was 26.38% lower than that in Scenario 2. Figure 9 indicates that congestion was likely to be less severe when demand was spread over a longer period of time as shown in Scenario 1.

To further explain the differences in congestion described in Figure 9, we analyzed the simulation results and made more comparison from two aspects, including highest crowd density identification and crowd degree.

**Figure 10:**
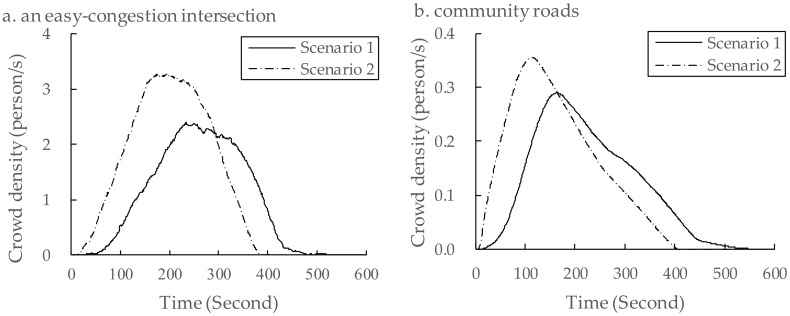
Crowd density identification of road sections. (**a**) The crowd density of residents on an easily-congested road intersection; (**b**) the crowding situation on the whole roads.

Firstly, Figure 10a shows the crowd density of residents on an easily-congested road intersection. It was found that the apparent time of peak density for Scenario 1 had shifted to the right of the crowd density for Scenario 2. Another example is Figure 10b. From this figure, it can be seen that the highest density time on the whole roads also appeared earlier in Scenario 2.

Secondly, Figure 10a and b also illustrate the degrees of crowd densities on an easily-congested road intersection and on the whole roads, respectively. The peak densities were overestimated by 36.77% and 22.74% in Scenario 2 when comparing with those of Scenario 1. Such overestimation reduces the accuracy of evacuation simulations and hinders the decision-making in evacuation management and optimization. Thus, from our point of view, pre-evacuation time estimation is of great importance in evacuation simulations, especially in the case of an urban residential community.

## 4. Discussion

With the analysis of the simulation result in Section 3.3, it can be judged that the pre-evacuation estimation can improve the estimation procedure of the start time, end time, changing flow rate and spatio-temporal crowd density. 

**Figure 11:**
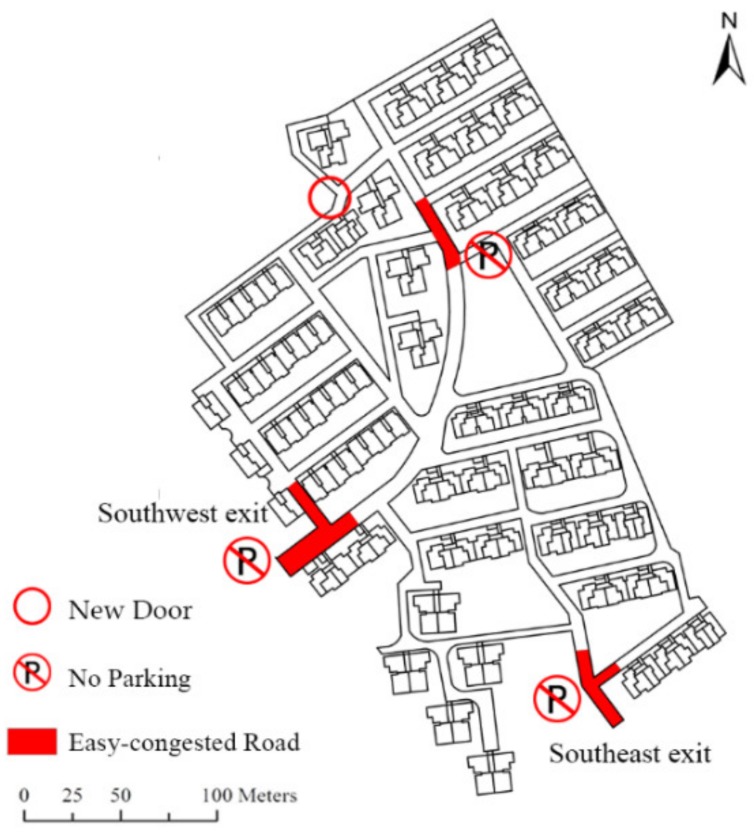
Easy-congested road sections in Luoshanqicun Community, Pudong New District, Shanghai, China.

In this study, we also tried to propose some evacuation management strategies and recommendations for emergency management after the case study: (1) Three easy-congested road sections (Figure 11) should be set as no-parking zones to prohibit car parking. This is of great significance for the safety and efficiency of an emergency evacuation. (2) Taking traffic dispersion measures at critical road sections. Guide signs or LED display screens which are designed to guide the residents and enable them to evacuate rationally should be placed at the major road sections. (3) Opening a new North exit. The new exit can relieve the road evacuation pressure of the southern region, effectively reducing the crowd degree in evacuation and decrease the total evacuation time.

Compared with other existing relevant research, although it is not the first evacuation simulation which includes the pre-departure evacuation time estimation component [26], the main contributions of this study are as follows: (1) The proposed method can predict short pre-evacuation times in emergency evacuation simulations in urban communities, which is more realistic than traditional simulation methods. (2) The pre-evacuation times of residents are predicted on the basis of questionnaires by the Random Forest (RF) algorithm and can get qualified prediction accuracy. (3) It integrates three-dimensional building evacuation with an outdoor pedestrian evacuation simulation process in an urban residential community. (4) A sensitivity analysis is integrated into the method to assess the extent to which variation in each key parameter produces corresponding variation in the estimation of the total evacuation time. (5) Based on the spatial mapping of the simulation results, some management strategies are proposed to improve management of an evacuation.

The method proposed in this study has some limitations in the following aspects. Firstly, this method was based on ultra-detailed data on residents and buildings, including the three-dimensional information of buildings and behaviors of residents. This may be a time-consuming and difficult task to simulate evacuation with ultra-detailed data in a large area. Moreover, the field survey may only cover a small percentage of residents for obtaining the data source to estimate the pre-evacuation time if the study area is large. Secondly, we made some simplified assumptions for conducting our method, which may have been different to a realistic condition. This study did not consider some complex conditions, including some common causes of delays such as reuniting separated family members during departure time, incomplete compliance and evacuation shadow, physical mobility limitations, etc. In the future, we will improve this method to simulate more complex evacuation behaviors on a larger spatial scale.

## 5. Conclusions

This study proposed a new method to address the evacuation problem of a vertical building evacuation followed by a small-scale horizontal pedestrian area evacuation, which integrated the pre-evacuation time estimation and made evacuation simulations in urban residential communities more realistic. The pre-evacuation times of residents were predicted by the Random Forest (RF) algorithm on the basis of residents’ pre-evacuation behavior data obtained by field surveys. The proposed method also involved a sensitivity analysis to estimate the sensitivity of different evacuation simulation parameters based on the one-at-a-time (OAT) method. The evacuation simulation results can be used to identify potential evacuation problems and help decision-making in evacuation management. To demonstrate the feasibility of this study, a case study was carried out in Luoshanqicun Community, Pudong New District, Shanghai, China. There are 51 residential buildings and more than 4000 residents in this community, which is a typical scale for a community. On this scale, it is also suitable for collecting sufficient survey data from the residents. We collected the residents’ pre-evacuation behavioral data from a field survey by means of questionnaires and simulated the evacuation process of all the residents in the study area. According to the simulation results, such as evacuation time estimation, flow rate calculation and crowd density estimation, it was found that the pre-evacuation times had significant impacts on the simulation procedure. The analysis results proved that the proposed method could get better simulation results than traditional simulation methods. On the basis of the simulation and analysis results, the potential evacuation problems in the study area were also discussed, and some evacuation management strategies were proposed to support emergency management. The case study illustrated the practicality of the proposed method. The method may be applied in other similar case studies with relevant population census data and investigations.

## Figures and Tables

**Table 1:** Major questions involved in the prediction model.

Question No.	Details
Input variables	
Q(1)	What is your gender?
Q(2)	What is your age?
Q(3)	What is your education level?
Q(4)	How many floors does your residential building have?
Q(5)	Which floor do you live in your residential building?
Q(6)	How many people do you live with?
Q(7)	Who do you live with?
Q(8)	How about your health condition?
Q(9)	Are you familiar with your community?
Q(10)	Do you have experiences in emergencies?
Output variables	How long will you take in the pre-evacuation period?
Y	

**Table 2:** Statistics of different age and gender groups in the questionnaires.

Age	Age Group	Male	%	Female	%	Total	%
0–13	Children	15	4.04	16	4.32	32	8.36
14–35	Young	67	17.73	72	18.98	139	36.71
36–64	Middle-aged	83	21.81	89	23.36	171	45.17
> 65	Elderly	18	4.71	19	5.05	37	9.76
Total	Total	183	48.29	196	51.71	379	100

**Table 3:** The description of pre-evacuation behaviors.

Class	Description
Evacuating immediately	Evacuating as soon as possible when recognizing emergencies.
Searching information	Reassessing the authenticity and criticality of emergencies by themselves.
Alerting	Alerting others to evacuation as soon as possible.
Collecting belongings	Collecting personal belongings before evacuation, including necessities, valuables and important documents.
Calling up all members	Calling up all members living together, especially the elderly and children.
Others	Any other pre-evacuation behaviors.

**Table 4:** Cases of input and output variables.

Case Number	Input Variables										Output Variable
	Q(1)	Q(2)	Q(3)	Q(4)	Q(5)	Q(6)	Q(7)	Q(8)	Q(9)	Q(10)	Y
1	1	1	2	1	1	6	5	1	3	2	2
2	2	1	2	2	1	3	4	1	2	2	3
3	1	3	5	2	1	4	5	1	3	1	2
4	2	1	2	2	2	5	5	1	3	2	1
5	1	1	2	2	2	3	4	1	3	2	3
6	1	1	2	4	4	6	5	1	3	2	4
7	2	1	2	2	1	4	4	1	3	2	1
8	1	1	2	3	1	6	5	1	3	2	2
9	2	2	5	4	4	3	4	1	2	2	2
10	2	2	6	2	1	6	6	1	2	2	2

Remark: The "case number" represents the sequence number of the case; Q(n) represents the *n*th question (input variable) listed in Table 1. In total there were 379 questionnaires, however, only 10 cases are listed here.

**Table 5:** The parameters of the prediction model (the RF algorithm).

Parameters	Value	Description
ntree	500	Number of trees
classwt	[1; 1; 1; 1]	Priors of classes
cutoff	[0.25; 0.25; 0.25; 0.25]	A vector of length equal to the number of classes
mtry	3	Number of predictors sampled for splitting at each node
replace	1	Sampling without replacement
nodesize	1	Minimum size of terminal nodes

**Table 6:** The predicted results of RF for multiple input variables and the respondents’ actual responses.

No.	Q(1)	Q(2)	Q(3)	Q(4)	Q(5)	Q(6)	Q(7)	Q(8)	Q(9)	Q(10)	Predicted Result (Time Interval)	Actual Response (Time Interval)
1	2	3	5	2	2	5	5	1	3	2	2	2
2	2	1	2	2	1	3	4	1	2	2	1	1
3	2	2	6	2	2	4	6	1	2	2	2	2
4	2	2	5	2	2	3	3	1	2	1	2	2
5	2	3	4	2	2	2	2	2	3	2	3	2
6	2	2	5	2	1	4	6	1	3	2	1	2
7	1	3	4	4	2	4	3	1	3	1	3	3
8	2	3	5	2	2	7	5	1	3	1	2	2
9	1	3	4	3	3	2	2	2	3	2	3	3
10	2	1	2	2	1	3	4	1	2	2	1	1

Remark: "No." represents the sequence number of residents in the Luoshanqicun Community; only 10 cases of data are shown in the table.

**Table 7:** Comparison of the prediction performance of the Random Forest and Back-Propagation (BP) neural network.

Class	Time Interval	Prediction Accuracy (%)	
		RF Algorithm	BP Neural Network
1	0–120s	92.86	50.00
2	120–300s	90.91	71.43
3	300–600s	71.43	89.74
4	>600s	50.00	0.00
Total		88.14	81.36

**Table 8:** Parameter setting of different sexes and age groups.

Category	Children	Young Female	Young Male	Middle-Aged Female	Middle-Aged Male	Elder Female	Elder Male
Walking speed (m/s)	1.19	1.27	1.32	1.2	1.25	1.08	1.1
Shoulder width (cm)	35	38	41	39.5	41.9	39	40.5
Acceleration time (s)	1.1	1.1	1.1	1.1	1.1	1.1	1.1
Distance to obstacles (m)	0.15	0.15	0.15	0.15	0.15	0.15	0.15
Comfortable distance (m)	0.08	0.08	0.08	0.08	0.08	0.08	0.08
Comfortable distance from queue density (person/m^2^)	4	4	4	4	4	4	4
Persist time (s)	1	1	1	1	1	1	1
Collision response time (s)	1.5	1.5	1.5	1.5	1.5	1.5	1.5

**Table 9:** A summary of the simulation results under two scenarios.

	Location	First In (s)	First Out (s)	Last Out (s)	Totally Pass (person)	Average Flow Rate (person/s)
Scenario 1	Southwest exit		47.3	483.6	1065	2.44
	Southeast exit		36.4	578.3	3603	6.65
	The whole road network	10.7		578.3	4668	
	Buildings		10.7	361.9	4668	0.28
Scenario 2	Southwest exit		40.5	482.2	1123	2.54
	Southeast exit		25.5	447.5	3545	8.4
	The whole road network	5.1		482.2	4668	
	Buildings		5.1	401	4668	0.77

## Appendix A

**Table A1:** Questionnaire on pre-evacuation behavior of community residents in an emergency.

Part I Basic Personal Information			
Index	Question	Options	
1	Gender	A. Male	
		B. Female	
2	Age	A. Below 13	
		B. 14–35	
		C. 36–64	
		D. Above 65	
3	Education Level	A. Primary or below	
		B. Secondary	
		C. High	
		D. Undergraduate	
		E. Postgraduate or above	
4	How many floors does your residential building have?	A. 1–3	
		B. 4–6	
		C. 7–9	
		D. Above 10	
5	Which floor do you live in your residential building?	A. 1–3	
		B. 4–6	
		C. 7–9	
		D. Above 10	
6	How many people do you live with?	A. 0	
		B. 1	
		C. 2	
		D. 3	
		E. 4	
		F. 5	
		G. 6 or above	
7	Who do you live with?	A. Spouse	
		B. Spouse and children	
		C. Parents	
		D. Parents, spouse and children	
		E. Friends	
8	How about your health condition?	A. Healthy	
		B. Unhealthy	
9	Are you familiar with your community?	A. Unfamiliar	
		B. Only familiar with the main exits	
		C. Familiar	
10	Do you have experiences in emergencies?	A. Yes	
		B. No	
**Part II The Behavior of the Personnel at the Initial Stage of the Emergency**			
**Index**	**Question**	**Options**	
11	Which actions will you take during the pre-evacuation process (multiple choices)?	A. Evacuating immediately	
		B. Searching information	
		C. Alerting	
		D. Collecting belongings	
		E. Calling up all members	
		F. Others	
12	How long will you take to do the actions which you selected in the previous question (Please give specific time if you select G)?	Searching information	A. 0–20 s
			B. 21–40 s
			C. 41–60 s
			D. 61–120 s
			E. 121–300 s
			F. 301–600 s
			G. Above 600 s (______s) *
		Alerting	A. 0–20 s
			B. 21–40 s
			C. 41–60 s
			D. 61–120 s
			E. 121–300 s
			F. 301–600 s
			G. Above 600 s (______s) *
		Collecting belongings	A. 0–20 s
			B. 21–40 s
			C. 41–60 s
			D. 61–120 s
			E. 121–300 s
			F. 301–600 s
			G. Above 600 s (______s) *
		Calling up all members	A. 0–20 s
			B. 21–40 s
			C. 41–60 s
			D. 61–120 s
			E. 121–300 s
			F. 301–600 s
			G. Above 600 s (______s) *
		Others	A. 0–20 s
			B. 21–40 s
			C. 41–60 s
			D. 61–120 s
			E. 121–300 s
			F. 301–600 s
			G. Above 600 s (______s) *
13	Considering the above questions, how long will you take in the pre-evacuation period?	A. 0–120 s	
		B. 120–300 s	
		C. 300–600 s	
		D. Above 600 s (______s) *	

* If this option is selected, the respondent needs to fill in the specific pre-evacuation time.
